# Genomic Plasticity and Molecular Mechanisms of Antimalarial Drug Resistance in *Plasmodium falciparum*: Implications for Treatment Failure and Genomic Surveillance

**DOI:** 10.1155/bmri/9296351

**Published:** 2026-10-09

**Authors:** Anmut Assemie

**Affiliations:** ^1^ Department of Biology Wachemo University Hossana Ethiopia wcu.edu.et

**Keywords:** genomic plasticity, genomic surveillance, malaria, *Plasmodium falciparum*

## Abstract

Malaria remains a major global public health challenge, in part because *Plasmodium falciparum* populations can acquire and disseminate reduced susceptibility to antimalarial drugs under selective pressure. This review synthesizes current evidence on the molecular and genomic mechanisms underlying antimalarial drug resistance and examines their implications for treatment efficacy and malaria control. First, the review examines genomic plasticity, including structural genome variation, copy‐number variation, recombination, epigenetic regulation, and transcriptional regulation, as sources of genetic and phenotypic variation relevant to drug susceptibility. Second, it summarizes established molecular determinants of resistance, including *kelch13* (*K13*) mutations associated with delayed parasite clearance following artemisinin treatment, *pfcrt* mutations associated with chloroquine resistance, *pfmdr1* copy‐number variation associated with altered susceptibility to mefloquine and related antimalarials, and *dhfr* and *dhps* mutations associated with sulfadoxine–pyrimethamine resistance. Third, the review considers the emergence and dissemination of resistance‐associated variants across parasite populations, emphasizing the contributions of population genomics and genomic surveillance to monitoring resistance patterns. Finally, recent applications of genomic epidemiology, whole‐genome sequencing, and artificial intelligence–assisted analytical approaches are discussed, with emphasis on their potential utility for resistance surveillance and evidence‐informed malaria control. Overall, integrating molecular, genomic, epidemiological, and evolutionary information provides a stronger framework for understanding antimalarial resistance and for supporting surveillance, treatment optimization, and malaria‐control strategies.

## 1. Introduction

Malaria remains one of the world′s most important infectious diseases and continues to pose a major global public health challenge despite more than a century of scientific advances and decades of coordinated malaria‐control efforts. According to the World Health Organization (WHO) World Malaria Report 2025, an estimated 282 million malaria cases and approximately 610,000 malaria deaths occurred worldwide in 2024, with about 95% of deaths occurring in the WHO African Region, predominantly among children under 5 years of age [1].

Historically, antimalarial drug resistance was often described as a sequence of geographically separated events. Chloroquine resistance, sulfadoxine–pyrimethamine failure, mefloquine resistance, and more recent artemisinin partial resistance illustrate successive episodes in which drug selection acted on parasite genetic variation. Genomic epidemiology has shown that resistance‐associated variants can arise independently, persist within populations, or disseminate through connected transmission networks [2–5].

*Plasmodium falciparum* populations exhibit genomic and regulatory plasticity, including copy‐number variation (CNV), structural genome variation, homologous recombination, gene amplification, epigenetic regulation, and transcriptional switching. These mechanisms contribute to genetic and phenotypic diversity and can influence responses to antimalarial drug pressure, host immunity, and other environmental conditions [6, 7]. Unlike simplified models of microbial resistance, *P. falciparum* adaptation is shaped by large parasite population sizes, standing genetic variation, recombination, CNV, and selection acting across geographically connected parasite populations. These processes can generate, maintain, and disseminate resistance‐associated variants across populations.

For the purposes of this review, we use the term “*Plasmodium*” resistome to describe the collective repertoire of resistance‐associated genes, mutations, CNVs, structural genomic changes, and regulatory mechanisms contributing to antimalarial drug resistance within and across parasite populations [4, 5].

Genomic epidemiology has demonstrated that resistance‐associated alleles and multidrug‐resistant *P. falciparum* lineages spread across national and continental boundaries through interconnected transmission networks driven by human mobility, cross‐border migration, and regional antimalarial drug pressure. Studies from the Greater Mekong Subregion have documented the expansion of artemisinin‐resistant and piperaquine‐resistant parasite lineages across Southeast Asia, while increasing genomic surveillance has identified the emergence and dissemination of *kelch13* mutations in Africa, highlighting the global connectivity of parasite populations [3, 5, 8–10].

The clinical implications of resistance extend beyond individual drug failures. Resistance‐associated variants can interact epistatically, and their phenotypic effects may depend on the broader parasite genetic background [11].

Thus, malaria control must account not only for drug discovery and program implementation but also for the evolutionary and population‐genomic processes that influence resistance emergence and dissemination. Understanding genomic plasticity, genetic variation, and population connectivity is important for interpreting changes in antimalarial susceptibility and for designing strategies intended to limit resistance.

This review synthesizes current insights into the genomic architecture, evolutionary dynamics, and systems‐level mechanisms of *Plasmodium* adaptation, reframing antimalarial drug resistance as a complex global evolutionary process driven by interconnected molecular pathways, population structure, and selective pressures rather than as a series of independent molecular events.

## 2. The Evolutionary Logic of *Plasmodium* Survival

At first glance, the malaria parasite *P. falciparum* appears evolutionarily constrained. Its complex life cycle requires both mosquito and human hosts, and its relatively compact genome reflects a high degree of specialization to parasitism [12, 13]. Nevertheless, *P. falciparum* has demonstrated substantial evolutionary resilience, persisting under repeated drug pressure, host immune selection, and public‐health interventions [14]. This resilience provides the evolutionary context for understanding the emergence and dissemination of antimalarial drug resistance.

*P. falciparum* adapts through the interaction of large parasite population sizes, repeated cycles of asexual replication, meiotic recombination within mosquito vectors, and genetic variation on which natural selection can act. During infection, extensive parasite replication provides opportunities for genetic variants to arise and increase in frequency under selective pressure [6]. In this evolutionary framework, drug exposure can favor variants that improve parasite survival, whereas recombination and population processes can influence their subsequent distribution.

### 2.1. Survival Through Predictable Adaptation

Drug resistance in *Plasmodium* can arise repeatedly in geographically separated parasite populations. Independent populations across Southeast Asia, Africa, and South America have acquired resistance‐associated variants in loci including *pfcrt*, *dhfr*, *dhps*, and *kelch13*, providing evidence of parallel or convergent evolution [2, 4]. These observations indicate that natural selection can repeatedly favor particular genetic solutions under similar drug‐selection pressures.

The repeated emergence of resistance‐associated variants suggests that some genomic pathways may be particularly accessible to selection under specific drug pressures. Consequently, resistance can emerge rapidly when pre‐existing variation or newly generated variants provide a fitness advantage under treatment. These observations should be interpreted as consequences of genetic variation and natural selection rather than as evidence of predetermined evolutionary trajectories.

### 2.2. Epistasis and the Architecture of Multidrug Resistance

Resistance‐associated variants can interact with the broader genetic background of the parasite. Epistatic interactions, in which the phenotypic effect of one allele depends on other genetic variants, may modify fitness costs and influence the expression or persistence of resistance‐associated phenotypes [11]. Such genetic backgrounds can contribute to differences in resistance phenotypes among parasite populations exposed to similar drug pressures.

These interactions indicate that resistance phenotypes may reflect combinations of resistance‐associated alleles and their genetic backgrounds rather than individual mutations alone. Persistence of particular combinations after changes in drug exposure may reflect selection, fitness effects, and population structure rather than a discrete form of evolutionary memory.

Epistatic interactions may also contribute to differences in the phenotypic effects of the same resistance‐associated mutation among parasite populations. The effect of a resistance variant can depend on accompanying genetic variation that modifies fitness or drug susceptibility.

### 2.3. Population Connectivity and the Dissemination of Resistance

Resistance‐associated alleles can persist within regional parasite populations, occur on different genetic backgrounds through recombination, and increase in frequency when corresponding selective pressures recur [5]. Human mobility, cross‐border transmission, and parasite movement can facilitate the dissemination of resistance‐associated variants among geographically connected populations.

Accordingly, changes in parasite genetic composition across transmission settings reflect the combined effects of mutation, recombination, natural selection, migration, and demographic processes. These processes can influence the persistence and dissemination of resistance‐associated variants as drug‐selection pressures change.

### 2.4. Evolutionary Flexibility and Resistance Emergence

The evolutionary success of *Plasmodium* reflects its capacity to maintain sufficient genetic and phenotypic variation to respond to changing selective environments. Genomic plasticity, recombination, antigenic diversity, and variation in mutational accessibility can contribute to this evolutionary flexibility [7]. These mechanisms provide potential substrates for selection, although their effects on resistance emergence depend on genetic background, drug exposure, and population processes.

Collectively, these evolutionary processes provide a framework for understanding the emergence and dissemination of antimalarial resistance. Genetic variation and natural selection influence the emergence of resistance‐associated phenotypes, whereas recombination, genetic background, and population connectivity can affect their persistence and distribution. The historical progression of major antimalarial resistance phenotypes and their molecular characterization is summarized in Figure 1.

**Figure 1:**
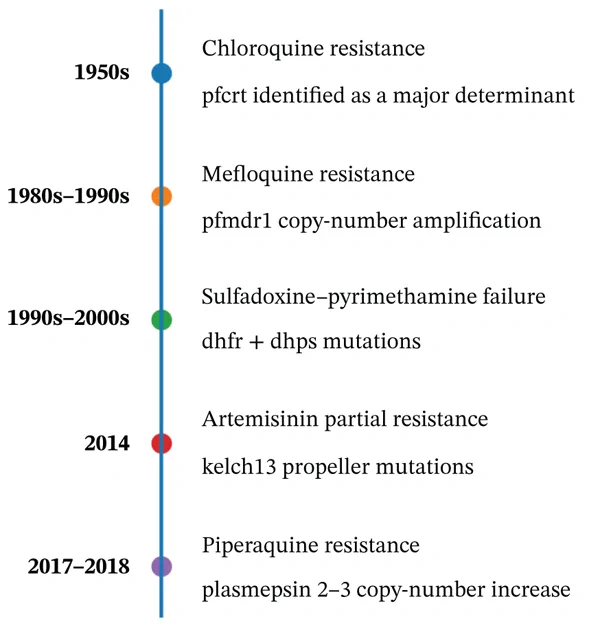
Selected milestones in the evolution and molecular characterization of antimalarial drug resistance in *P. falciparum*. The timeline links major resistance episodes with subsequently characterized molecular determinants [15–19].

## 3. Genomic Plasticity and the Molecular Evolution of Resistance

Genomic plasticity provides molecular mechanisms through which *P. falciparum* can generate and regulate genetic and phenotypic variation under changing environmental conditions. Resistance‐associated variants occur in several well‐characterized loci, including *pfcrt*, *pfmdr1*, *dhfr*, *dhps*, and *kelch13*, while copy‐number and structural variation can further modify parasite phenotypes and drug susceptibility [17, 20].

These forms of genomic and regulatory variation provide mechanisms through which parasite phenotypes can change under environmental and selective pressures. Structural variation, epigenetic regulation, transcriptional control, and posttranscriptional processes can modify gene dosage or expression and thereby influence parasite phenotypes [21–24].

### 3.1. CNV and Structural Genome Remodeling

CNV is a form of structural genomic variation in which specific DNA segments are duplicated or deleted, resulting in differences in gene copy number among parasite isolates. Because gene dosage can influence gene expression, CNVs can alter parasite phenotypes without requiring changes in the nucleotide sequence of individual genes. In *P. falciparum*, CNVs have been associated with variation in drug susceptibility and other parasite phenotypes, providing an important mechanism of genomic plasticity [11].

Amplification of specific loci has been associated with altered drug susceptibility in *P. falciparum*. Examples include *pfmdr1*, which is associated with altered susceptibility to mefloquine and other antimalarial drugs; *gch1*, which has been associated with antifolate susceptibility; and the plasmepsin 2–3 locus, which has been associated with piperaquine resistance in particular parasite genetic backgrounds [11, 25]. These examples illustrate how changes in gene dosage can modify parasite phenotypes without requiring point mutations within the affected genes.

Whole‐genome analyses indicate that *Plasmodium* genomes contain recombination hotspots, subtelomeric regions, and chromosomal regions susceptible to structural rearrangements [6]. These features can facilitate duplications, deletions, and segmental rearrangements, contributing to variation in genes involved in parasite metabolism, transport, and virulence.

### 3.2. Epigenetic Regulation and Transcriptional Switching

Epigenetic regulation refers to changes in gene expression that occur without alterations in the underlying DNA sequence. In *P. falciparum*, these processes involve chromatin remodeling, histone modifications, nucleosome positioning, and other regulatory mechanisms that influence transcriptional activity [21]. Epigenetic regulation can therefore contribute to phenotypic plasticity by allowing changes in gene expression without requiring permanent changes in DNA sequence.

The *var*, *rifin*, and *stevor* multigene families, which contribute to immune evasion and cytoadherence, are regulated through transcriptional switching mechanisms that allow parasites to alter gene‐expression states without changes to the underlying DNA sequence [22]. Some of these expression states can persist through successive cell divisions, demonstrating the contribution of epigenetic regulation to phenotypic variation.

Epigenetic regulation may influence parasite responses to environmental stress and drug exposure, including transient changes in gene expression and phenotype; however, the contribution of such regulatory states to clinically relevant drug resistance remains uncertain [22, 26].

### 3.3. Noncoding RNAs (ncRNAs) and Posttranscriptional Control

ncRNAs are RNA molecules that are transcribed but not translated into proteins, and they can regulate gene expression at both transcriptional and posttranscriptional levels. In *P. falciparum*, these include long ncRNAs, antisense RNAs, and circular RNAs, which have been implicated in the regulation of mRNA stability, translation, chromatin organization, and developmental processes [23, 24, 27]. Their regulatory effects provide an additional mechanism through which parasite gene expression can vary without changes to protein‐coding DNA sequences.

Stage‐specific ncRNAs may contribute to developmental transitions, metabolic remodeling, and stress responses by modifying gene expression and protein output. Such regulation may be relevant during antimalarial drug exposure, although the specific contribution of individual ncRNAs to clinically relevant drug resistance requires further investigation.

Together, structural variation, epigenetic regulation, transcriptional switching, and ncRNA‐mediated control demonstrate that genomic plasticity operates through multiple genetic and regulatory mechanisms. These mechanisms can generate or modify phenotypic variation and thereby influence parasite responses to environmental and drug‐selection pressures. These relationships are integrated in Figure 2, which summarizes how selective pressures, genomic and regulatory variation, population processes, phenotypes, and surveillance interact within an evolutionary framework.

**Figure 2:**
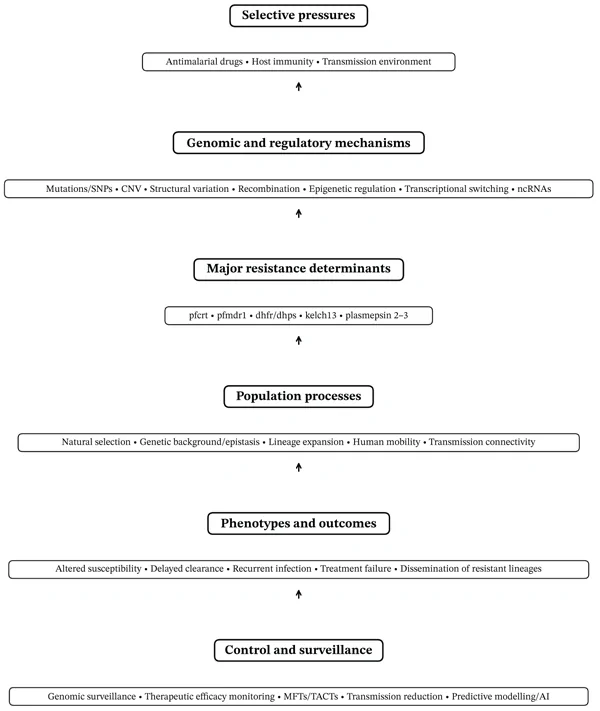
Evolutionary framework of antimalarial drug resistance in *P. falciparum*. Selective pressures act on genomic and regulatory variation; resistance‐associated determinants interact with genetic background and population processes; resulting phenotypes influence clinical and epidemiological outcomes; and surveillance and intervention strategies provide feedback for resistance management [28–31].

Collectively, CNV, structural genome remodeling, epigenetic regulation, transcriptional switching, and ncRNA‐mediated control illustrate the multiple molecular mechanisms contributing to genomic and regulatory plasticity in *P. falciparum*. These mechanisms can influence phenotypic variation and drug susceptibility, although their effects on resistance depend on genetic background, drug exposure, and population‐level selection. The population distribution and dissemination of resistance‐associated determinants are considered in the following section.

## 4. Architecture of the Global *Plasmodium* Resistome

In this review, the term “global *Plasmodium* resistome” is used as a conceptual framework to describe the geographically distributed collection of resistance‐associated genetic determinants and their occurrence across parasite populations. Rather than representing a discrete biological entity, the framework integrates information on resistance‐associated variants, their genetic backgrounds, and their geographic distribution to examine how resistance emerges and spreads among connected populations.

*P. falciparum* populations are structured geographically but remain connected through parasite transmission and human movement. Population‐genomic studies have shown that resistance‐associated variants can emerge independently in different regions or spread from established resistant populations into new transmission settings [4, 32]. The resulting geographic distribution of resistance reflects the combined effects of local drug‐selection histories, parasite population structure, recombination, migration, and transmission intensity. Thus, understanding antimalarial resistance requires consideration not only of individual resistance determinants but also of their distribution among interconnected parasite populations.

The population‐genomic structure of antimalarial resistance can be considered across three related dimensions: genetic interactions among resistance‐associated loci, geographic variation in resistance profiles, and the movement of resistant parasite lineages between populations. Table 1 summarizes these components and emphasizes how local selection and population connectivity contribute to differences in the distribution and persistence of resistance‐associated variants.

**Table 1:** Global *Plasmodium* resistome architecture: major components, evolutionary mechanisms, and implications for the emergence and spread of antimalarial drug resistance.

Component	Definition	Mechanism	Implication	Examples	References
Polygenic resistance networks	Networks of interacting alleles across multiple loci	Epistatic interactions stabilize fitness costs	Resistance persists even under combination therapy	*kelch13* + background mutations	[11]
Geographic stratification	Region‐specific patterns of resistance alleles	Local selection pressures + drug histories shape allele frequency	Emergence of localized resistant populations	Southeast Asia: artemisinin and partner‐drug resistance; Africa: emerging resistance‐associated variants with heterogeneous phenotypic evidence	[4]
Human‐mediated spread	Dissemination of resistance alleles via travel or migration	Parasite populations mix and recombine globally	Resistant alleles exported before clinical failure	Geographically connected parasite populations	[5, 33, 34]

### 4.1. Polygenic Resistance Networks

Resistance phenotypes in *P. falciparum* can reflect interactions among established resistance‐associated loci and the broader parasite genetic background. Key loci, including *pfcrt*, *pfmdr1*, *dhfr*, *dhps*, and *kelch13*, may therefore occur within distinct combinations of background variants that modify their phenotypic effects [11]. This population‐genomic context helps explain why the same resistance‐associated allele may have different frequencies or phenotypic consequences among parasite populations.

Genome‐wide analyses indicate that genetic background can influence the expression, fitness effects, and persistence of resistance‐associated variants [2]. Such interactions contribute to population‐specific resistance profiles and may affect whether newly emerging variants increase in frequency or remain at low prevalence. Consequently, characterization of resistance requires analysis of linked genomic variation rather than reliance on individual marker loci alone.

### 4.2. Geographic Stratification of Drug Escape Mutations

Despite connectivity among parasite populations, resistance‐associated variants show substantial geographic structure. Regional resistance profiles reflect differences in antimalarial drug use, parasite population history, transmission intensity, and the genetic backgrounds in which resistance‐associated variants occur [3, 4]. Southeast Asia has been a major region for the emergence and dissemination of multidrug‐resistant *P. falciparum* lineages, including parasites carrying artemisinin‐ and partner‐drug‐resistance‐associated determinants [4, 15]. In African populations, *kelch13* resistance‐associated variants have emerged independently, demonstrating that resistance can arise under region‐specific selective conditions rather than solely through introduction from other regions [2].

Population‐genomic analyses can distinguish independent emergence from geographic spread by comparing the genomic backgrounds surrounding resistance‐associated variants [5]. Such analyses are particularly useful for determining whether similar resistance phenotypes in different regions reflect repeated local emergence or the expansion of related parasite lineages.

### 4.3. Human Mobility and the Spread of Resistant Lineages

Human mobility and cross‐border transmission can connect geographically separated *P. falciparum* populations and facilitate the movement of resistant parasite lineages. Whole‐genome sequencing has been particularly informative for reconstructing these transmission pathways. The expansion of the KEL1/PLA1 lineage across the Greater Mekong Subregion illustrates how a multidrug‐resistant lineage can disseminate across national boundaries, while genomic studies in Africa have identified locally emerging *kelch13* variants associated with delayed parasite clearance [3, 5, 8–10, 35].

Genomic epidemiology can also identify relationships among resistant parasite populations and provide evidence of historical or recent movement between transmission settings [5].

Overall, the geographic distribution of antimalarial resistance reflects the interaction of genetic background, local selection, population structure, and parasite movement between transmission settings. Viewing resistance within this population‐genomic framework helps distinguish independent emergence from lineage dissemination and provides a basis for interpreting regional differences in resistance patterns.

## 5. Host–Parasite Molecular Arms Race

Host immune pressure is a major selective force shaping the biology and persistence of *P. falciparum*. During infection, parasite survival and persistence are influenced by interactions with innate and adaptive immune responses that affect parasite density, duration of infection, tissue distribution, and transmission [36, 37]. Parasite traits that alter antigenic expression, erythrocyte adhesion, and sequestration can reduce immune‐mediated clearance and influence disease manifestations. These interactions also contribute to reciprocal selection between parasite traits and host genetic variation, including erythrocyte polymorphisms and immune‐response loci [38, 39].

### 5.1. Immune Evasion and Antigenic Diversification

Immune‐mediated selection is strongly influenced by antigenic variation in *P. falciparum*. The *var* gene family encodes PfEMP1, a major surface protein involved in cytoadherence and interactions with the host immune system. Parasites can express different members of the *var* repertoire during infection, generating antigenic variation that can alter recognition by host antibodies [22].

Switching among PfEMP1 variants can modify the antigenic phenotype of infected erythrocytes and influence the duration of infection by reducing the effectiveness of variant‐specific antibody responses [40]. Changes in PfEMP1 expression can also affect cytoadherence phenotypes and tissue distribution, linking antigenic variation to both immune evasion and sequestration.

Other multigene families, including *rifin* and *stevor*, contribute additional antigenic and erythrocyte‐interaction diversity and may influence immune modulation, rosetting, and parasite persistence [41]. Together, these variant surface‐protein systems increase phenotypic diversity during infection and provide multiple targets through which host immunity can shape parasite population composition.

### 5.2. Cytoadherence, Sequestration, and Pathogenic Persistence

Cytoadherence and sequestration are central features of blood‐stage *P. falciparum* infection. Infected erythrocytes expressing PfEMP1 can bind endothelial receptors, including ICAM‐1, CD36, EPCR, and chondroitin sulfate A (CSA), reducing splenic clearance of infected cells and promoting sequestration in specific vascular compartments [42].

The distribution and receptor‐binding properties of PfEMP1 variants influence where infected erythrocytes accumulate and are associated with clinical manifestations such as cerebral and placental malaria [43].

## 6. Mechanisms of Antimalarial Failure

Artemisinin‐based combination therapies (ACTs) remain central to the treatment of *P. falciparum* malaria, but their effectiveness can be reduced when parasites acquire decreased susceptibility to artemisinin derivatives and/or their partner drugs. Artemisinin partial resistance is primarily associated with mutations in the *kelch13* propeller domain and is characterized by delayed parasite clearance [8, 9]. Reduced susceptibility to partner drugs, including piperaquine, mefloquine, and lumefantrine, can further increase the risk of recurrent infection and treatment failure when combined with impaired artemisinin susceptibility [2, 10, 35].

### 6.1. Artemisinin Partial Resistance and Delayed Clearance

Artemisinin partial resistance is primarily associated with mutations in the *kelch13* propeller domain and is expressed clinically as delayed clearance of parasitemia following artemisinin treatment [15]. The phenotype is most evident during the early ring stage of the parasite life cycle, when resistant parasites exhibit increased survival following transient artemisinin exposure. Consequently, partial resistance does not necessarily produce immediate treatment failure but can prolong parasite persistence and increase the opportunity for surviving parasites to contribute to subsequent transmission.

The phenotypic effect of *kelch13* mutations depends on the genetic background in which they occur, with additional loci contributing to parasite fitness and resistance expression [2]. This genetic dependence helps explain variation in the clinical and epidemiological consequences of *kelch13*‐associated resistance among parasite populations.

### 6.2. Partner‐Drug Failure in ACTs

Reduced susceptibility to ACT partner drugs can convert delayed parasite clearance into recurrent infection or overt treatment failure. Different partner drugs are associated with distinct molecular determinants. For example, amplification of the *plasmepsin 2–3* region is associated with reduced piperaquine susceptibility, whereas increased *pfmdr1* copy number is associated with reduced mefloquine susceptibility [11, 25]. Changes in *pfmdr1* and related loci can also influence susceptibility to other ACT partner drugs, including lumefantrine.

When partial artemisinin resistance occurs together with reduced susceptibility to a partner drug, the combined effect can decrease the effective activity of the ACT regimen. Surviving parasites may therefore persist after treatment and contribute to recrudescence or subsequent transmission. The clinical significance of partner‐drug resistance depends on the specific drug combination, parasite genetic background, treatment exposure, and local resistance profile.

This distinction is important because ACT failure is not determined by a single resistance marker. Instead, treatment outcome reflects the interaction between the susceptibility phenotype of the parasite and the pharmacological activity of the drugs used. Specific resistance loci and their mechanisms are summarized in Table 2.

**Table 2:** Major *Plasmodium* drug resistance‐associated loci: genomic mechanisms, geographic distribution, and polygenic interactions underlying antimalarial resistance.

Gene	Associated drug(s)	Mechanism of resistance	Geographic hotspots	Polygenic interactions	References
kelch13	Artemisinin	Increased survival of early ring‐stage parasites following transient artemisinin exposure	Greater Mekong Subregion, Cambodia, East Africa	Requires a permissive multilocus background	[2, 15]
pfcrt	Chloroquine	Altered digestive‐vacuole transport and drug efflux	Africa, Asia, South America	Epistatic with *pfmdr1*	[3, 11]
pfmdr1	Mefloquine, Lumefantrine	Gene copy‐number amplification can alter susceptibility to mefloquine and other antimalarial drugs	Southeast Asia, East Africa	Epistatic with *kelch13*	[11, 25]
dhfr	Pyrimethamine	Point mutations altering folate pathway enzymes	Africa, Asia	Synergistic with *dhps*	[2, 44, 45]
dhps	Sulfadoxine	Point mutations reducing sulfonamide binding	Africa, Asia	Synergistic with *dhfr*	[44, 45]
plasmepsin2–3	Piperaquine	Segmental gene amplification increases hemoglobin digestion	Cambodia, Greater Mekong Subregion	Coselected with *kelch13*	[25]

## 7. Emerging Surveillance and Predictive Genomics

Surveillance of antimalarial resistance increasingly requires approaches that can detect genetic change before it is reflected in widespread clinical treatment failure. Conventional therapeutic efficacy studies remain essential for measuring treatment outcomes, but genomic surveillance can provide earlier information on the emergence, frequency, and geographic distribution of resistance‐associated variants [3]. Integration of whole‐genome sequencing, targeted genotyping, population‐genomic analysis, and epidemiological data can therefore strengthen resistance monitoring and support timely interpretation of emerging threats [46, 47]. The principal value of predictive genomics is not to replace clinical surveillance, but to complement it by identifying genomic signals that may warrant closer therapeutic and epidemiological investigation.

### 7.1. Genomic Epidemiology and Resistance Forecasting

Whole‐genome sequencing and targeted genomic approaches can provide high‐resolution information on the population structure, genetic diversity, and geographic distribution of *P. falciparum*. Genomic epidemiology can be used to distinguish locally emerging resistance‐associated variants from those introduced through parasite movement and to characterize the genetic backgrounds in which resistance determinants occur [3, 5]. These analyses complement conventional surveillance by providing information on lineage relationships and transmission connectivity that may not be apparent from clinical data alone.

### 7.2. Artificial Intelligence (AI) in Malaria Surveillance

AI and machine‐learning approaches can enhance malaria surveillance by integrating epidemiological, environmental, entomological, and genomic data. These methods have shown potential for malaria forecasting, spatial risk mapping, and surveillance. However, their application requires high‐quality, representative datasets and appropriate validation across different geographic and epidemiological settings [48].

AI‐based approaches should therefore be viewed primarily as analytical tools that can augment genomic surveillance rather than as autonomous predictors of treatment failure. Their practical value will depend on the quality and geographic representativeness of training data, the availability of linked genotype–phenotype and clinical outcome datasets, and the ability to validate predictions across transmission settings. Integrating machine‐learning outputs with established molecular markers and epidemiological surveillance may provide a more robust basis for identifying emerging resistance patterns.

## 8. Evolution‐Aware Strategies to Disrupt Antimalarial Resistance

Evolution‐informed antimalarial strategies aim to reduce the probability that resistant parasites will emerge, persist, or spread under treatment pressure. Rather than relying solely on the replacement of drugs after resistance becomes clinically established, these approaches consider parasite genetic diversity, cross‐resistance, fitness effects, and transmission dynamics when selecting and deploying interventions [16, 26, 49, 50]. The objective is to increase the genetic and epidemiological barriers to resistance while maintaining effective treatment.

Several approaches have been proposed within this framework, including multiple first‐line therapies (MFTs), triple artemisinin‐based combination therapies (TACTs), resistance‐breaking drug cycling, and interventions that reduce transmission of resistant parasites [51–54]. Genomic surveillance can complement these strategies by identifying changes in resistance‐associated variants and informing decisions about treatment policy. The major evolution‐aware strategies and their potential roles in delaying the emergence and spread of antimalarial drug resistance are summarized in Table 3.

**Table 3:** Evolution‐aware antimalarial strategies: genomic mechanisms, evolutionary principles, and Their potential roles in delaying the emergence and spread of drug resistance.

Strategy	Evolutionary principle	Primary rationale	Potential contribution	References
Multiple first‐line therapies (MFT)	Diversified drug selection	Reduces sustained selection by a single regimen	May slow resistance fixation	[51]
Triple ACTs (TACTs)	Increased barrier to simultaneous resistance	Combines multiple antimalarial activities	May delay emergence of resistance	[22]
Resistance‐breaking drug cycling	Collateral sensitivity	Uses changes in susceptibility between drugs	May reduce selection for particular resistant genotypes	[52, 53]
Transmission‐blocking interventions	Reduction of parasite transmission	Limits onward transmission of resistant parasites	May reduce dissemination	[54]
Genomic surveillance	Early detection of resistance‐associated change	Monitors allele frequencies, haplotypes, and population structure	Supports timely intervention	[3, 5]
AI‐assisted modeling	Integration of complex datasets	Identifies patterns and candidate predictors in genomic and epidemiological data	May improve risk assessment	[55]
Gametocytocidal interventions	Reduction of transmissible parasite stages	Decreases contribution of treated infections to onward transmission	May limit spread of resistant parasites	[56, 57]

### 8.1. Resistance‐Breaking Drug Cycling

Resistance‐breaking drug cycling is based on the possibility that resistance to one antimalarial may alter parasite susceptibility to another drug. Conventional rotation assumes that resistance‐associated fitness costs will reduce the frequency of resistant variants when the corresponding drug is withdrawn. However, the persistence of resistance alleles and interactions among loci may limit this effect [11]. Drug cycling can therefore be designed around documented collateral‐sensitivity relationships rather than simple temporal replacement of one drug by another.

Where reciprocal susceptibility patterns are sufficiently consistent, alternating drugs may reduce the selective advantage of particular resistant genotypes and potentially restore susceptibility to previously compromised compounds [52]. However, the effectiveness of such strategies is likely to depend on local parasite population structure, resistance genotype frequencies, drug exposure, and the stability of collateral‐sensitivity relationships. Consequently, drug cycling should be supported by molecular and therapeutic surveillance rather than implemented as a uniform strategy across settings.

### 8.2. Next‐Generation Combination Therapies

Combination therapy can also be optimized to increase the barrier to treatment failure. In addition to pharmacokinetic compatibility and independent antimalarial activity, candidate combinations should be evaluated for cross‐resistance, resistance‐selection potential, and the likelihood that a parasite can survive exposure to all components. Triple ACTs (TACTs) represent one approach intended to increase the number of concurrent adaptations required for survival and have shown potential for delaying resistance emergence in settings where partner‐drug susceptibility is preserved [58, 59].

Future combinations may incorporate compounds with complementary molecular targets or activity against different parasite developmental stages. However, the evolutionary advantage of any combination will depend on drug exposure, adherence, parasite genetic background, and the prevalence of pre‐existing resistance. Combination design should therefore be evaluated using integrated pharmacological, genomic, phenotypic, and clinical evidence rather than genetic mechanism alone.

### 8.3. Targeting Transmission Bottlenecks

Reducing transmission provides an additional means of limiting the spread of resistant parasite lineages. Interventions that target gametocytes, block parasite development in the mosquito, or reduce vector‐mediated transmission can decrease the number of parasites entering subsequent transmission cycles [54]. In principle, reducing transmission can also reduce opportunities for resistant parasites to recombine and disseminate between populations.

The principal value of transmission‐blocking interventions in resistance management is therefore complementary to drug treatment: they can reduce the onward propagation of parasites that survive antimalarial exposure. Their impact on resistance evolution will depend on intervention coverage, transmission intensity, parasite population structure, and the extent to which resistant parasites contribute to transmission. Combining transmission‐reducing measures with effective case management may therefore help limit the geographic spread of resistance while reducing disease transmission.

## 9. Research Gaps and Future Directions in *Plasmodium* Genomic Adaptation

Despite substantial progress in characterizing antimalarial resistance, important uncertainties remain concerning the genetic and regulatory determinants of parasite adaptation. Current genomic surveillance is particularly limited in its ability to resolve rare variants, structural variation, noncoding regions, transient phenotypes, and the contribution of genetic background to resistance‐associated phenotypes. In addition, genomic data remain unevenly distributed geographically, limiting the ability to compare resistance dynamics across transmission settings.

Future research should therefore prioritize functional interpretation of genomic variation, longitudinal sampling, improved representation of under‐sampled parasite populations, and integration of genomic data with phenotypic and clinical measurements. These priorities would help distinguish variants that are merely associated with resistance from those that contribute directly to resistance or treatment outcomes. They also provide a basis for improving surveillance and evaluating evolution‐informed interventions. The major research priorities are outlined below.

### 9.1. Decoding the Full Adaptive Genome

Although canonical resistance loci such as *kelch13*, *pfcrt*, and *pfmdr1* remain important for surveillance, genome‐wide studies indicate that additional loci and genetic backgrounds may influence parasite responses to drug pressure [60, 61]. A major research gap is therefore to determine the functional contribution of candidate variants, including cryptic and compensatory changes that may not be captured by marker‐based surveillance. Functional genomics, combined with phenotype‐linked population genomic data, could help distinguish causal determinants from variants that are merely associated with resistant parasite populations.

Resolving these relationships would improve interpretation of genomic surveillance data and provide a stronger basis for identifying resistance‐associated variants that warrant further clinical and epidemiological investigation.

### 9.2. Linking Epigenetics, ncRNAs, and Drug Tolerance

Beyond DNA sequence variation, regulatory and epigenetic processes may contribute to parasite phenotypic variation and responses to environmental or drug‐associated stress [62]. However, the extent to which these processes contribute directly to clinically relevant drug tolerance, rather than representing transient responses to cellular stress, remains incompletely resolved. Recent analyses of noncoding variation and chromatin‐associated features also suggest that regulatory regions may influence parasite phenotypes and warrant further investigation [63].

Future studies should therefore integrate longitudinal transcriptomic, epigenomic, and genetic measurements with drug‐response phenotypes to determine whether specific regulatory states are reproducibly associated with altered susceptibility. Functional validation will be particularly important for distinguishing regulatory associations from causal mechanisms. This work could clarify the contribution of nongenetic variation to treatment response without assuming that transient tolerance necessarily progresses to fixed genetic resistance.

### 9.3. Global Resistome Dynamics and Human Connectivity

A major limitation of current knowledge is the uneven geographic coverage of genomic surveillance and the resulting uncertainty about how resistance‐associated variants are introduced, maintained, and selected in different parasite populations. Genomic surveillance in Rwanda has provided evidence of diverse resistance‐associated variation and ongoing parasite evolution, illustrating the value of longitudinal sampling for characterizing local resistance dynamics [64].

Future studies should expand longitudinal and geographically representative sampling and combine genomic data with information on transmission intensity, treatment exposure, and where appropriate, parasite movement. Such datasets could help distinguish locally selected variants from introduced variation and improve understanding of how resistance‐associated alleles become established within different epidemiological settings.

### 9.4. Evolution‐Informed Therapeutics and Implementation Science

Several resistance‐management strategies discussed in Section 8 require further evaluation under routine program conditions. Important questions include whether resistance‐breaking drug cycling, optimized combination therapies, and transmission‐reducing interventions provide measurable benefits when applied alongside existing malaria‐control programs. Their effectiveness may also depend on treatment adherence, drug availability, transmission intensity, local resistance patterns, and health‐system capacity.

Translational and implementation research should therefore evaluate these strategies using clinical studies, therapeutic efficacy monitoring, epidemiological data, and operational assessments. Particular attention should be given to determining how genomic and epidemiological information can be incorporated into treatment‐policy decisions in endemic and resource‐limited settings [64].

### 9.5. Integrating AI and Predictive Modeling

AI and machine‐learning methods provide opportunities to integrate genomic, epidemiological, and other malaria‐related datasets, but their application to antimalarial resistance requires further validation [63]. Important limitations include incomplete or geographically biased datasets, limited linkage between genotype and phenotype, model overfitting, and uncertainty about whether predictions developed in one parasite population or transmission setting remain reliable elsewhere.

Future research should therefore prioritize geographically diverse training datasets, external validation, transparent reporting of uncertainty, and comparison of model predictions with longitudinal genomic, phenotypic, and clinical observations. The practical value of AI should ultimately be assessed by whether predictive models improve the identification and prioritization of resistance threats rather than by predictive performance alone.

## 10. Conclusion

Antimalarial drug resistance in *P. falciparum* is shaped by interactions among genetic variation, genomic plasticity, natural selection, recombination, genetic background, and parasite population structure. Established resistance‐associated determinants, including *pfcrt*, *pfmdr1*, *dhfr*, *dhps*, *kelch13*, and copy‐number changes involving relevant loci, demonstrate that reduced drug susceptibility can arise through multiple molecular and genomic mechanisms. However, the phenotypic and clinical significance of individual variants depends on parasite genetic background, drug exposure, transmission setting, and interactions among resistance‐associated loci.

An evolution‐informed understanding of resistance therefore complements, rather than replaces, conventional malaria surveillance and control. Molecular and genomic surveillance can provide information on the distribution and frequency of resistance‐associated variants and can help identify changes in parasite populations that warrant further epidemiological or phenotypic investigation. Integration of genomic data with therapeutic, epidemiological, and clinical information may improve the evidence base for treatment policy and resistance monitoring.

Future malaria‐control strategies should prioritize validated combination therapies, appropriate treatment use, continued monitoring of drug efficacy, and geographically representative genomic surveillance. Genomic and epidemiological data can also support the evaluation of emerging resistance patterns and help guide timely public‐health responses. AI and predictive modeling may provide additional analytical capacity, but their practical utility will depend on data quality, external validation, appropriate model interpretation, and linkage between genomic markers and clinically relevant phenotypes.

Overall, antimalarial resistance should be considered a dynamic population‐level process rather than a collection of isolated mutations. Continued integration of molecular biology, evolutionary genetics, genomic epidemiology, clinical surveillance, and vector‐control programs will be important for understanding resistance trajectories and for developing sustainable strategies to preserve the effectiveness of available antimalarial interventions.

## Funding

No funding was received for this manuscript.

## Ethics Statement

The author has nothing to report..

## Consent

The author has nothing to report.

## Conflicts of Interest

The author declares no conflicts of interest.

## Data Availability

All the data used to support the findings of this research were included in the manuscript.
